# Single amino acid supplementation in aminoacidopathies: a systematic review

**DOI:** 10.1186/1750-1172-9-7

**Published:** 2014-01-13

**Authors:** Danique van Vliet, Terry GJ Derks, Margreet van Rijn, Martijn J de Groot, Anita MacDonald, M Rebecca Heiner-Fokkema, Francjan J van Spronsen

**Affiliations:** 1 Department of Metabolic Diseases, Beatrix Children’s Hospital, University Medical Center Groningen, University of Groningen, Groningen, the Netherlands; 2 Dietetic Department, Birmingham Children’s Hospital, Birmingham, United Kingdom; 3 Laboratory of Metabolic Diseases, Department of Laboratory Medicine, University Medical Center Groningen, University of Groningen, Groningen, the Netherlands

**Keywords:** Aminoacidopathies, Inborn errors of metabolism, Single amino acid supplementation, Dietary management, Amino acid mixture, Organic acidurias

## Abstract

Aminoacidopathies are a group of rare and diverse disorders, caused by the deficiency of an enzyme or transporter involved in amino acid metabolism. For most aminoacidopathies, dietary management is the mainstay of treatment. Such treatment includes severe natural protein restriction, combined with protein substitution with all amino acids except the amino acids prior to the metabolic block and enriched with the amino acid that has become essential by the enzymatic defect. For some aminoacidopathies, supplementation of one or two amino acids, that have not become essential by the enzymatic defect, has been suggested. This so-called single amino acid supplementation can serve different treatment objectives, but evidence is limited. The aim of the present article is to provide a systematic review on the reasons for applications of single amino acid supplementation in aminoacidopathies treated with natural protein restriction and synthetic amino acid mixtures.

## Introduction

Inborn errors of amino acid metabolism or aminoacidopathies are a group of rare and diverse disorders, in total affecting about 1 in 1000 humans worldwide [1]. These disorders can be subdivided in organic acidurias, urea cycle defects, transport defects of urea cycle intermediates, and remaining aminoacidopathies. Clinical phenotypes are highly variable, ranging from asymptomatic to life-threatening metabolic decompensation already at neonatal age, encompassing slow deterioration of mental capacities at later age [1].

At present, dietary management is the mainstay of treatment for most aminoacidopathies. Dietary treatment aims to prevent accumulation of the substrates and associated metabolites to toxic levels, and to restore deficiencies of the enzymatic products [2]. This can be accomplished by natural protein restriction, combined with protein substitution with all amino acids except for the amino acids prior to the metabolic block and enriched with the amino acid that has become essential by the enzymatic defect [3,4]. Also, additional supplementation of one or two single amino acids may be required for other purposes.

This so-called single amino acid (SAA) supplementation is especially important to overcome a deficiency of the amino acid that has become essential due to the enzymatic defect. Clinically, deficiencies of specific amino acids may result in skin (hair and nail) problems, poor growth, and developmental delay [5-7]. Biochemically, such deficiencies might be detected by studying amino acid concentrations in plasma. An alternative strategy to detect amino acid deficiencies is the indicator amino acid oxidation method [8], but this method should be used for research purposes rather than for clinical practice.

The main objective of this article is to present a systematic review on SAA supplementation in aminoacidopathies. We focus on objectives other than to overcome a deficiency of the amino acid that has become essential by the enzymatic defect. Such treatment objectives include:

A. Prevention of a deficiency of a specific amino acid that has not become essential by the enzymatic defect.

B. Prevention of toxic accumulation of specific substrates prior to the metabolic block, either by reducing synthesis or increasing excretion.

C. Competition with toxic agents for entry into target organs, especially the brain.

To our best knowledge, we identified all aminoacidopathies treated with natural protein restriction and synthetic amino acid mixtures for which additional SAA supplementation has been reported. The rationale underlying the treatment strategies and reported results were determined, while taking into account the levels of evidence.

## Methods

### Search strategy

We conducted a literature search on PubMed and EMBASE without date limits up to 30th of October, 2012. In PubMed, the Medical Subject Headings (MesH) terms used included (“Amino Acids/therapeutic use”[Mesh]) AND ("Amino Acid Metabolism, Inborn Errors"[Mesh] OR "Amino Acid Transport Disorders, Inborn"[Mesh] OR "Metabolism, Inborn Errors"[Mesh:NoExp]). In EMBASE, EMTREE tools used included ('amino acid'/exp/dd_dt,dd_ad,dd_ct) AND (('disorders of amino acid and protein metabolism'/exp) OR ('inborn error of metabolism'/de). The search term subheadings dd_dt, dd_ad, and dd_ct refer to drug therapy, administration and dosage, and clinical trial respectively. Additional searches were performed on 10th of June 2013 to identify possible newly added articles (published in 2012 or 2013 only) that were not yet indexed for either MesH terms or EMTREE tools. In these additional searches, (isovaleric acidemia AND glycine), (methylmalonic acidemia AND (isoleucine OR valine)), (“propionic acidemia” AND (isoleucine OR valine OR glycine)), (glutaric aciduria type 1 AND (arginine OR homoarginine OR ornithine)), (maple syrup urine disease AND (isoleucine OR valine OR norleucine)), (phenylketonuria AND (tryptophan OR threonine OR glutamine OR glutamate OR asparagine)), (tyrosinemia type 1 AND phenylalanine), ((“gyrate atrophy” OR “ornithine aminotransferase deficiency”) AND (lysine OR proline)), (guanidinoacetate methyltransferase deficiency AND ornithine), and (homocystinuria AND (cysteine OR cystine OR arginine)) were entered as free text for both databases. Language limits for all searches were set at English and German.

### Study selection

**Figure 1:**
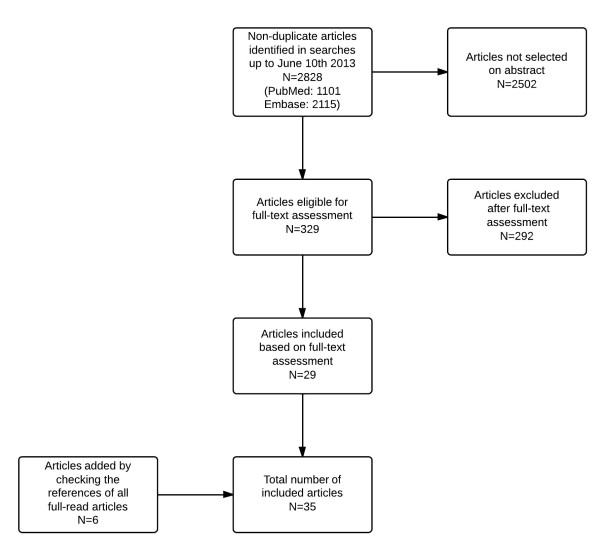
Flow-chart of the reviewing process.

First, titles and/or abstracts of all identified non-duplicate references were screened to select eligible studies. Eligibility criteria included: 1) involving an aminoacidopathy which is treated (among else) with natural protein restriction and a synthetic amino acid mixture; 2) possibly referring to SAA supplementation. Then, full-text articles of the selected references were retrieved and read independently to assess whether the inclusion criteria were met. Inclusion criteria were: 1) SAA treatment regimens are clearly defined; 2) SAA treatment effects are clearly defined. Articles only addressing the application of SAA supplementation to restore the concentrations of the amino acid that has become essential by the enzymatic defect were excluded, as were abstracts and conference proceedings, and research performed in animal models that are not based on a genetic defect. The reference lists of all full-read articles were reviewed to identify additional eligible studies. Results of the reviewing process are outlined in Figure 1.

## Results

Table 1 presents an overview of all relevant aminoacidopathies for which SAA supplementation has been described, including the treatment strategy and the level of evidence. This table includes only the aminoacidopathies for which severe natural protein restriction and an amino acid mixture devoid of the offending precursor amino acids or an essential amino acid supplement is a generally accepted treatment. More detailed information about the results of SAA supplementation in these aminoacidopathies can be found in Additional file 1. In addition, three aminoacidopathies (hyperammonaemia-hyperornithinaemia-homocitrullinuria syndrome, lysinuric protein intolerance, and nonketotic hyperglycinemia), for which combined natural protein restriction and an essential amino acid mixture is more controversial, are worth discussion with respect to SAA supplementation. We will now continue by addressing these SAA supplementation regimens for each aminoacidopathy individually.

**Table 1:** Suggested applications of SAA supplements in different aminoacidopathies

**Disorder**	**Treatment objective**			**Level of evidence**	
	**A**	**B**	**C**		
**IVA**		Glycine		4-5	
**MMA**	Isoleucine + Valine			5	
**PA**		Glycine		5	
	Isoleucine + Valine			5	
**GA-I**			Arginine	3b	animal
			Homoarginine	-	animal
			Ornithine	-	animal
**MSUD**			Isoleucine + Valine	-	
			Norleucine	-	animal
	Isoleucine + Valine			4-5	
**PKU**		Glutamine		5	
		Glutamate		5	
		Asparagine		5	
		Threonine		3b	
			MAIB	-	animal
			AIB	-	animal
			NB	-	animal
			Norleucine	-	animal
	Tryptophan			4	
	Glutamine			4	
**HT1**	Phenylalanine			4	
**OAT deficiency**		Lysine		4	
	Proline			4	
**GAMT deficiency**		Ornithine	Ornithine	5	
**HCU**			Arginine	5	
		Cysteine		-	
	Arginine*	Arginine*	Arginine*	1b	

Therapeutic objective A) correction of amino acid deficiency; B) prevention of toxic accumulation of specific substrates prior to the metabolic block; C) competition with toxic agents for entry into target organs.

MAIB: N-methyl-aminoisobutyrate; AIB: 2-aminoisobutyrate; NB: 2-aminonorbornane

*Treatment objective is unclear.

### Isovaleric acidemia

Isovaleric acidemia (McKusick 243500) is an inherited defect of leucine catabolism, caused by isovaleryl-CoA dehydrogenase deficiency. This deficiency results in the accumulation of isovaleryl-CoA and its metabolites, including isovaleric acid (IVA). Under stable conditions, detoxification proceeds by alternate pathways in glycine- and carnitine conjugates, including N-isovalerylglycine (IVG), which can be easily excreted by the kidneys. However, after protein intake and/or periods of catabolism [9], capacity of this alternative pathway detoxification does not suffice. This results in ketoacidosis or, sometimes, even in coma.

In the case of glycine supplementation treatment, leucine-loading tests showed higher urinary excretion of IVG [10-12] and related metabolites [11,12], less increased plasma IVA concentrations [10,11,13], and no vomiting [10]. In the case of acute metabolic decompensation, often precipitated by illness, glycine supplementation has been described to result in a decline of plasma IVA concentrations, concomitant with an increase in urinary IVG excretion [13-15], followed by a neurological and haematological response after one to two weeks of treatment [14,15].

As episodes of metabolic decompensation are difficult to predict, chronic management with glycine to prevent such episodes has been investigated [16-20]. Several studies support lower doses for maintenance treatment than for acute management, in order to prevent side-effects due to hyperglycinemia [16-18]. At oral dosages of 300 mg/kg/d, plasma glycine concentrations up to 1680 μM have been observed, concomitant with increased lethargy and ataxia [17,18]. In contrast, Naglak et. al. (1988) did not report on any encephalopathic side-effects of glycine dosed at 600 mg/kg under constant leucine restriction, although plasma glycine concentrations at this dosage even reached 2547 ± 591 μM [16]. Alternatively, to circumvent possible encephalopathic side-effects or to increase treatment effectiveness, carnitine administration has been proposed either as monotherapy or in addition to glycine supplementation [12,17-20]. However, considering the scope of the present review, evidence on carnitine supplementation in isovaleric acidemia is not discussed in further detail. To conclude, glycine supplementation has been shown to be effective both in acute and chronic management of individual IVA patients (level 4–5), but encephalopathic side-effects have been reported at plasma glycine concentrations > 1000 μM.

### Methylmalonic acidemia

Methylmalonic acidemia (MMA; McKusick 251000) is caused by methylmalonyl-CoA mutase deficiency. To reduce accumulation of methylmalonyl–CoA and associated metabolites, dietary restriction of precursor amino acids (valine, isoleucine, methionine, and threonine) and sometimes also odd-chain fatty acids is used. In addition, fasting should be avoided to prevent endogenous catabolism. Under this treatment regimen, some patients suffered from severe acrodermatitis enteropathica like skin lesions (despite adequate blood zinc concentrations) which, in some cases, even culminated in sepsis and death [21]. Especially isoleucine deficiency has been associated with these skin lesions [6,22-24]. In MMA patients on dietary treatment, isoleucine and valine deficiencies are regularly observed [25].

SAA supplementation with isoleucine (48–340 mg/d) and valine (68–170 mg/d) to prevent essential amino acid deficiencies and associated skin lesions has become routine clinical practice in some centers without any reported adverse effects [26]. Still, defining optimal dietary treatment is very difficult [27], exemplified by the fact that not only metabolic crises still occur if offending precursor amino acids are elevated due to endogenous catabolism, but offending precursor amino acids can become overly restricted as well. To conclude, additional isoleucine and valine supplementation to prevent deficiencies in MMA patients has become routine clinical practice in some centers. Currently, the level of evidence is 5. So far, its effects and possible side-effects have not been investigated.

### Propionic acidemia

Propionic acidemia (PA; McKusick 232000) is characterized by propionyl-CoA carboxylase deficiency, blocking the BCAA catabolic pathway one step earlier than in MMA. Dietary treatment is very similar to that used in MMA, with restriction of natural protein and sometimes also odd-chain fatty acids, as well as supplementation of a synthetic amino acid mixture devoid of valine, isoleucine, methionine, and threonine being reported. In addition, fasting should be avoided. In the past, it has been incorrectly assumed that leucine should be restricted as well, leading to growth restriction without any benefit on PA symptomatology [28].

As in other inborn errors of amino acid metabolism, various SAA supplementation treatment strategies have been proposed. The most widely suggested one is the use of isoleucine and valine supplementation, comparable with the application described for MMA [29]. As in MMA, deficiencies of both isoleucine and valine are often observed [25,30]. In addition, Scholl-Bürgi et al. showed that blood isoleucine and valine concentrations, in contrast to almost all other amino acids, did not correlate with age [31]. To prevent a relative isoleucine deficiency, the use of routine isoleucine supplementation (100 mg/d) has been reported [32].

A different SAA treatment includes supplementation of glycine to promote conjugation of propionyl CoA and tiglyl CoA, and thereby to stimulate urinary excretion. This may seem contra intuitive, as hyperglycinaemia is observed in nearly all PA patients [30]. Blood glycine concentrations in PA patients, however, have been found to correlate with blood bicarbonate concentrations [33]. Furthermore, the affinity of propionate to conjugate with either glycine or carnitine has been hypothesized to be pH-sensitive, glycine being favoured during periods of ketoacidosis. Although urinary excretion of propionylglycine and tiglylglycine indeed increased on glycine supplementation (two doses of 200 mg/kg), this was complicated by arterial hyperammonemia [34]. It was therefore concluded that glycine supplementation was not desirable for PA management. However, it could well be that the arterial hyperammonemia was due to the increased nitrogen load rather than the glycine supplementation itself, as protein intake had been restricted earlier to 0.9 g/kg/day for 1.0 g/kg/day was not tolerated. To conclude, the effect of isoleucine and valine supplementation in PA management has not been investigated, although preventive isoleucine supplementation is currently applied in some centers (level 5). Glycine supplementation clearly was not effective (level 5).

### Glutaric aciduria type I

Glutaric aciduria type I (GA-I; McKusick 231670) is caused by glutaryl-CoA dehydrogenase deficiency, which is required for lysine and tryptophan oxidation. Especially, elevated brain concentrations of glutaric acid, due to increased cerebral lysine catabolism, correlate with GA-I symptomatology: acute encephalopathic crises, often typically precipitated by mild infections. Current management includes a protein restricted diet with a lysine-free and low-tryptophan amino acid mixture, and carnitine administration [35].

To reduce brain lysine concentrations even further without inducing non-brain lysine deficiencies, supplementation of substrates competing with lysine for brain uptake by the y+ transporter has been suggested as an additional treatment modality [36-38]. Indeed, homoarginine (a homologue of L-arginine, synthesized from lysine) or arginine supplementation in a GA-I mouse model showed promising results, whereas ornithine supplementation resulted in increased mortality rates, similar to high-protein diet exposure [39]. Administration of homoarginine reduced brain lysine and glutaric acid accumulation, and increased survival in a GA-I mouse model [40]. In addition, arginine supplementation decreased glutaric acid and 3-hydroxyglutaric acid concentrations in both brain and liver. As system y^+^ transport is only expressed at the BBB, the inhibition of hepatic metabolite accumulation by arginine supplementation is possibly mediated by a different transport mechanism. Reduced lysine concentrations in cerebral and hepatic mitochondria of GA-I mice receiving arginine supplementation are indicative of the fact that the human mitochondrial ornithine carrier 1 might be such a mediating transporter [41].

In GA-I patients, administration of an arginine-fortified amino acid supplement biochemically resulted in reduced plasma lysine concentrations and urinary 3-hydroxyglutarate excretion, compared to historical data on GA-I patients receiving conventional lysine restricted dietary regimens. This was accompanied by reduced calculated brain lysine, and increased calculated brain arginine influx. However, arginine fortification did not improve growth [42]. In patients receiving either one of two amino acid supplements, fortified with different amounts of arginine, no significant correlations were found between arginine intake and plasma lysine-to-arginine ratios or neurological outcome [43].

Recommendations for arginine fortification in both chronic and outpatient sick-day management have been established based on aforementioned studies. In chronic management, dietary intake of lysine and arginine of 65–85 mg/kg/day and 100–150 mg/kg/day are suggested, aiming at a ratio of dietary lysine/arginine intake of 0.5-0.8. For outpatient sick-day management, recommended dietary lysine intake is even lower (30–35 mg/kg/day), whereas recommended dietary arginine intake is even higher aiming at a dietary lysine/arginine intake of 0.15-0.20 (mg:mg) [42]. To conclude, homoarginine and arginine supplementation decreased brain lysine and glutaric acid concentrations in a GA-I mouse model. In patients, arginine fortification showed reduced calculated brain lysine (level 3b), but no clinical improvement (level 3b), and the effect on actual brain lysine and glutaric acid concentrations still needs to be investigated.

### Maple syrup urine disease

Maple syrup urine disease (MSUD; McKusick 231670) is an inherited defect in BCAA catabolism caused by deficient branched-chain α-ketoacid dehydrogenase. Although all BCAA accumulate, leucine accumulation is considered to be particularly toxic and associated with cerebral manifestations. Episodes of metabolic decompensation mainly occur by endogenous protein catabolism, as observed in normal postpartum state of neonates or precipitated by mild infections or physiologic stress in older patients. Dietary management aims to restrict BCAA intake and to prevent endogenous protein catabolism. Classically, MSUD amino acid formula was thus designed to contain no BCAA. However, limited and strongly regulated supplementation of isoleucine and valine has been suggested both in chronic and acute dietary management [44].

By indicator amino acid oxidation measurement, Riazi et al. estimated the mean total BCAA requirement for MSUD patients to be 45 mg/kg/d (leucine 17.3 mg/kg/d; isoleucine 14.6 mg/kg/d; and valine 13.1 mg/kg/d) [45]. Especially chronic deficiency of isoleucine may result in acrodermatitis enteropathica-like skin eruptions [46-48]. At present, no acrodermatitis enteropathica-like syndromes have been reported in centers in which supplementation of isoleucine and valine as well as regular monitoring of blood BCAA concentrations has become routine clinical practice [44,49].

During episodes of acute metabolic decompensation, supplementation of isoleucine and valine could serve other treatment strategies. First, isoleucine and valine may become limited factors for protein synthesis, thereby inducing increased blood leucine concentrations due to increased proteolysis. To prevent this, supplementation of isoleucine and valine has been suggested [50,51]. Second, supplementation of isoleucine and valine has been suggested to compete with leucine for brain uptake and thereby to counteract the encephalopathic effects of excessive brain leucine accumulation [44]. Such a mechanism has been shown for the non-physiological amino acid norleucine in an animal model of MSUD [52,53].

Recent management guidelines offer special attention to isoleucine and valine supplementation in both chronic and acute management [44,49] and emphasize on regular monitoring of BCAA in blood. In chronic management, supplementation of isoleucine and valine should be targeted at blood molar ratios of leucine/isoleucine = 2 and leucine/valine ≥ 0.5 respectively. To maintain these ratios, mean valine supplementation has been found to decrease during the first 3 years of life (11.5 mg/kg/d to 5.8 mg/kg/d), whereas mean isoleucine supplementation remained relatively stable (4.4 mg/kg/d to 5.5 mg/kg/d) [49]. During outpatient catabolic management, isoleucine and valine supplementation should be increased to 15–30 mg/kg/d, whereas inpatient catabolic management should include IV administration of isoleucine and valine at 20–120 mg/kg/d [49]. However, IV single valine and isoleucine solutions are not available in every country for use with MSUD patients. Research in MSUD patients, rats, and MSUD cell models showed that, of all BCAA, leucine is most toxic to the brain [54-56]. Nonetheless, cerebral toxicity from (chronically) elevated blood isoleucine and/or valine concentrations cannot be excluded based on the evidence currently available, and safe upper limits for blood isoleucine and valine have not yet been established. To conclude, isoleucine and valine supplementation in MSUD patients resolved dermatitis (level 4–5), but preventive treatment has not been studied. Moreover, isoleucine and valine supplementation during acute metabolic decompensation decreased blood leucine concentrations (level 5), whereas the effect on brain leucine concentrations still remains to be investigated.

### Phenylketonuria

Phenylketonuria (PKU; McKusick 261600) is caused by deficiency of phenylalanine hydroxylase, resulting in excessively elevated blood phenylalanine concentrations and normal to slightly decreased blood tyrosine concentrations. Especially the elevated blood phenylalanine concentrations have been associated with PKU symptomatology, including severe mental retardation, developmental delay, seizures, and psychiatric problems. Dietary management includes restriction of natural protein and a phenylalanine-free amino acid supplement. Although early initiation of this diet has abolished development of severe mental retardation and epilepsy, mild cognitive impairments as well as neuropsychological deficits still occur. Besides additional supplementation of tyrosine, different alternative SAA treatments have been reported. Apart from the very early but unsuccessful ideas about glutamine supplementation [57-59], the following suggestions on SAA supplementation have been proposed.

Threonine supplementation has been suggested to decrease blood phenylalanine concentrations. This concept was based on the observations that blood threonine concentrations were inversely related to blood phenylalanine concentrations in PKU patients [60] and that threonine administration in rats decreased blood phenylalanine concentrations exclusively [61]. Indeed, threonine supplementation (50 mg/kg/d; approximately 60% of unsupplemented threonine intake) in PKU patients reduced both blood phenylalanine concentrations as well as urinary phenylalanine excretion [62], possibly due to competition with phenylalanine for facilitated transport at the gut-blood barrier [63].

In the 1970s, tryptophan supplementation has been proposed to restore a possible tryptophan deficiency in brain. The experimental data originate from a rat model treated with phenylalanine and the phenylalanine hydroxylase inhibitor dl-p-chlorophenylalanine [64]. In this pharmacological model, tryptophan supplementation partly corrected impairments in the swim maze and of conditioned shock avoidance as well as decreased brain serotonin and 5-hydroxyindoleacetic acid concentrations. However, this rat model is now considered invalid to reflect PKU, as dl-p-chlorophenylalanine also inhibits tryptophan hydroxylase [65]. In two late-diagnosed PKU patients, supplementation of tryptophan (100 mg/kg/d) has been reported to increase serotonin metabolite concentrations in cerebrospinal fluid without influencing blood phenylalanine concentrations. Also, increased vigilance was observed in the one patient that showed abnormal vigilance when untreated [66].

Recently, administration of 2-aminoisobutyrate and non-physiological amino acids such as DL-norleucine, 2-aminonorbornane, and N-methyl-aminoisobutyrate acting as inhibitors for various brain amino acid transporters have been shown to reduce brain phenylalanine concentrations up to 56% in PKU mice [67]. To conclude, threonine supplementation in PKU patients decreased blood phenylalanine concentrations in a single study (level 3b), while tryptophan supplementation seemed to have a positive effect in two late-diagnosed and untreated patients (level 4).

### Tyrosinemia type I

Hereditary tyrosinemia type I (HT1; McKusick 276600) is caused by a deficiency of fumarylacetoacetase. This enzyme is responsible for the conversion of fumarylacetoacetate to fumarate and acetoacetate, the last step in tyrosine catabolism. Untreated, the enzymatic block results in accumulation of extremely toxic metabolites (including maleylacetoacetate, fumarylacetoacetate, succinylacetoacetate, and succinylacetone) causing liver damage. As a consequence of the liver damage, markedly elevated blood tyrosine (phenylalanine and methionine) concentrations occur. The principal treatment is the administration of 2-(2-nitro-4-trifluoromethylbenzyl)-1,3-cyclohexanedione (NTBC) to prevent accumulation of these toxic tyrosine metabolites by inhibiting tyrosine catabolism at an earlier step. Additional dietary management includes natural protein restriction supplemented with an amino acid mixture devoid of tyrosine as well as phenylalanine, as 27-41% of phenylalanine has been shown to be converted to tyrosine in the first 5–8 hours after intake [68,69]. Targeting the complications of this treatment regimen, two different types of single amino acid supplementation have been described.

Firstly, phenylalanine supplementation has been suggested to restore its potential deficiency [70]. With dietary restriction of both tyrosine and phenylalanine, very low blood phenylalanine concentrations have been reported [70,71], that may be improved by supplementing with additional phenylalanine [70]. Concerns have been raised that phenylalanine deficiency in HT1 would limit protein synthesis, and thereby impair cognitive outcome and growth [70,71]. However, no data are available on the safe lower limits of blood phenylalanine concentrations, which, theoretically, can be an age-dependent parameter, related to maintenance of protein synthesis to facilitate (brain) development and growth. On the other hand, phenylalanine supplementation may limit natural tyrosine tolerance by increased conversion of phenylalanine to tyrosine.

Secondly, in rats, threonine supplementation has been shown to partly prevent the ocular lesions caused by tyrosine toxicity [72-75]. Although the underlying mechanism is still unknown, it has been suggested that blood tyrosine concentrations decrease if tyrosine administration is combined with threonine [73,74]. Therefore, it can be hypothesized that threonine could protect against ocular lesions by lowering blood tyrosine concentrations either by competition with tyrosine and/or phenylalanine for uptake at the gut-blood barrier or in the renal tubules, or by promoting tyrosine oxidation in the liver [75]. However, as threonine supplementation has also been shown to partly prevent ocular lesions with NTBC administration without reducing blood tyrosine concentrations, the positive effects of threonine do not seem to be solely due to a reduction of hypertyrosinemia [75]. To conclude, additional phenylalanine supplementation in HT1 patients clearly increased the otherwise very low blood phenylalanine concentrations (level 4), but the possible clinical effects remain to be investigated. Moreover, threonine supplementation showed positive effects in hypertyrosinemic rats on ocular lesions, but no clinical studies have been conducted yet.

### Ornithine aminotransferase deficiency

Ornithine aminotransferase (OAT) deficiency or gyrate atrophy (GA; McKusick 258870) is an inherited deficiency of ornithine aminotransferase. Using pyridoxal-phosphate as a cofactor, the enzyme is responsible for the reversible conversion of ornithine and α-ketoglutarate to pyrroline-5-carboxylate and glutamate. Clinically, GA is mostly characterized by a slowly progressive loss of vision, culminating in blindness by the fifth decade of life. However, a neonatal presentation with acute hyperammonemia is also recognized [76]. Biochemically, the enzyme deficiency results in 10–20 times elevated blood ornithine concentrations. Although some patients respond to pyridoxine administration, the cornerstone of treatment is an arginine restricted diet. In practice, this treatment includes restriction of natural protein with supplementation of a synthetic amino acid mixture devoid of arginine.

Different SAA supplementation regimens have been investigated for OAT deficiency. Firstly, lysine supplementation (10–15 g/d) has been shown to decrease blood ornithine concentrations by increasing its urinary excretion [77,78]. This is probably due to the fact that ornithine and arginine share a common renal transport system with lysine and cyst(e)ine. Whether this treatment could slow or prevent loss of vision, or could increase protein tolerance, has not been investigated.

Secondly, proline supplementation has been hypothesized to restore a proline deficiency [79,80]. Ornithine catabolism is considered important for proline synthesis especially in the retinal pigment epithelium, where OAT activity is ten times higher compared to the liver [81]. Moreover, oral ornithine loading in GA patients could not increase blood proline concentrations as in normal subjects, suggesting that proline indeed becomes an essential amino acid in GA [80]. In an *in vitro* model of human retinal pigment epithelial cells, administration of proline could prevent the cytotoxic effects of OAT deficiency [82]. In GA patients, proline supplementation was found to minimize chorioretinal deterioration (in 3 of 4 patients) and even improve vision in 1 patient [79]. To conclude, lysine supplementation in GA patients reduced blood ornithine concentrations by increasing its urinary excretion (level 4), and proline supplementation seemed to have a positive effect on vision (level 4). However, further evidence is required.

### Guanidinoacetate methyltransferase deficiency

Guanidinoacetate methyltransferase (GAMT; McKusick 601240) deficiency is an inherited disorder of creatine synthesis. Clinically, untreated GAMT deficiency mainly results in expressive language impairments, extrapyramidal movements, epilepsy, autistic and self-injurious behaviour, and developmental delay. Biochemically, the enzymatic block prevents creatine to be synthesized from guanidinoacetate (GAA). Hence, the disorder is characterized by accumulation of GAA and deficiency of creatine. Treatment includes creatine supplementation in order to restore cerebral creatine levels [83]. GAA is considered to be toxic. To prevent accumulation, arginine restriction, administration of sodium benzoate, and ornithine supplementation have been proposed [84-86].

Arginine (combined with glycine) is the precursor of GAA. Ornithine may decrease the conversion of arginine to GAA [85] and reduce tubular arginine reabsorption, as both amino acids use the same dibasic amino acid transporter [87], while sodium benzoate removes glycine. Combined arginine restriction and ornithine supplementation (100 and 400 mg/kg/d) has been reported to decrease blood arginine as well as GAA concentrations [86,88]. Decreased arginine intake without ornithine supplementation does not have that effect [84]. Additional ornithine supplementation has been hypothesized to enhance the GAA lowering effect of dietary arginine restriction [86]. The exact ornithine dose needed may probably be between 600 and 800 mg/kg/d [83,89]. To conclude, ornithine supplementation in combination with arginine restriction may be helpful in decreasing blood arginine concentrations in GAMT deficiency patients (level 5).

### Homocystinuria

Homocystinuria (HCU; McKusick 263200) is an inherited deficiency of cystathionine β-synthase (CBS). Using pyridoxal-phosphate as a cofactor, the enzyme is involved in the transsulfuration of homocysteine to form cystathionine. Biochemically, HCU is primarily characterized by increased blood homocysteine and methionine concentrations. The elevated homocysteine concentrations are associated with clinical features, including ectopia lentis, mental retardation, dental anomalies, osteoporosis, behavioral problems, and arachnodactyly. Some patients respond very well to pyridoxine treatment. If not, dietary management is indicated including natural protein restriction, an amino acid mixture devoid of methionine, and supplementation of folate. In addition, different SAA supplementation regimens have been proposed.

In daily practice, cysteine is added to all L-amino acid supplements (30–50 mg of cysteine per g of protein equivalent). Besides restoring a deficiency of this amino acid, cysteine has been hypothesized to reduce blood homocysteine concentrations. However, to the best of our knowledge, no study has investigated the possible homocysteine-lowering effect of cysteine supplementation in CBS-deficient patients. Four main theories have been postulated for the possible mechanisms underlying this effect. As summarized by Kawakami et al., cysteine may 1) remove homocysteine from its protein-bound form to a low-molecular weight form, facilitating increased urinary clearance; 2) decrease homocysteine formation from methionine; and 3) increase remethylation of homocysteine to form methionine [90]. In support of the first hypothesis, cysteine has been shown to decrease the percentage of homocysteine bound to protein as well as the total homocysteine concentration in rats [90]. Both effects were only observed in rats fed a low protein and low methionine diet [90]. In contrast to the first hypothesis, a fourth hypothesis states that additional cysteine supplementation (in case of total blood cysteine concentrations <170 μmol/L) may reduce free plasma homocysteine concentrations [91]. In HCU patients with blood cystine concentrations <170 μmol/L, the homocysteine free/bound ratio was found to increase, possibly to maintain the total aminothiol free/bound ratio (thiol redox) at a constant level. Normally, this thiol redox is determined by the ratio of free/bound cysteine and is kept relatively constant [91].

Arginine has been proposed to be supplemented for two reasons: 1) to decrease endothelial dysfunction [92], and 2) to compete with renal homocysteine reabsorption [93]. Although the exact mechanism of vascular damage in HCU is not well understood, reduced nitrogen oxide (NO) probably plays a central role and increased concentrations of asymmetric dimethylarginine (ADMA), an endogenous NO synthase inhibitor, have been found. Arginine supplementation could counteract the effects of elevated ADMA concentrations [92]. Regarding its second treatment objective, arginine glutamate infusion has been reported to markedly increase urinary homocysteine and homocysteine-cysteine disulphide excretion. However, blood homocysteine concentrations remained unchanged, whereas blood cysteine, lysine, arginine, and ornithine concentrations increased [93]. To conclude, besides the restoration of a cysteine deficiency, cysteine supplementation in HCU patients may have additional effects that should be investigated. Moreover, arginine supplementation has been found to decrease endothelial dysfunction (level 1b), but the underlying mechanism is not fully understood.

### Hyperammonaemia-hyperornithinaemia-homocitrullinuria syndrome

Hyperammonaemia-hyperornithinaemia-homocitrullinuria (HHH; McKusick 238970) syndrome is an inherited defect of the ornithine transporter 1 located on the inner mitochondrial membrane. This transporter facilitates ornithine influx and citrulline efflux. Accordingly, the defect underlying HHH syndrome results in cytosolic ornithine accumulation and intramitochondrial ornithine deficiency as well as citrulline accumulation. Protein restriction is the main treatment. In some centers, an essential amino acid mixture, designed for urea cycle disorders, is prescribed according to clinical tolerance. A variety of SAA supplementation regimens have been investigated, including the amino acids ornithine, arginine, citrulline, lysine, and proline in HHH syndrome [94-98].

Supplementation with ornithine has been suggested, but ornithine hydrochloride rather than ornithine supplementation seems to result in a decrease of blood ammonia concentrations [94-96], while long-term (exceeding a few years) or high-dose (inducing blood concentrations exceeding 600 μmol/L) ornithine has been associated with retinal toxicity [97].

A study comparing the effects of ornithine, arginine or citrulline supplementation in two HHH patients suggested that citrulline is probably the best SAA to supplement for chronic HHH management [96,98], but a low protein diet only could be more important for chronic management. Supplementation of SAA would then be reserved for periods of metabolic decompensation [96]. Lysine or proline supplementation has also been proposed for HHH treatment, but the rationale for these hypothesized SAA treatments has not been fully defined. Neither lysine nor proline has been shown to be beneficial [96]. To conclude, of all SAA supplementation regimens investigated in HHH patients, citrulline supplementation was found to be most effective (level 4), but such supplementation should probably be reserved for periods of acute metabolic decompensation only.

### Lysinuric protein intolerance

Lysinuric protein intolerance (LPI; McKusick 222700) is an inherited defect in the y^+^LAT1 transporter, involved in the transport of the dibasic amino acids lysine, arginine, and ornithine in the intestine, kidney, and liver. Most patients develop a strong protein aversion. Incidental protein intake characteristically results in hyperammonemia, caused by a secondary dysfunction of the urea cycle due to arginine and ornithine deficiency. Additional clinical manifestations include growth retardation, osteoporosis, hepatosplenomegaly, pulmonary alveolar proteinosis, renal involvement, and hematological abnormalities. Biochemically, LPI is mainly characterized by markedly increased urinary arginine, ornithine, and lysine excretion and thereby reduced concentrations of these amino acids in blood. The mainstay of treatment includes natural protein restriction to prevent hyperammonemia, while also providing sufficient essential amino acids to maintain normal development and growth. To this purpose, sometimes, an essential amino acid mixture is added as in HHH [99]. Since the first description of LPI in 1965, different approaches on additional SAA supplementations have been suggested [100-112].

The most common approach is citrulline supplementation (0.5-1.1 mmol/kg/d), for which the rationale is to restore urea cycle functioning [100-102]. Clinically, long-term citrulline supplementation diminishes protein aversion [101,103,104], increases growth rate [101,103,104], improves hair quality [103,104], and diminishes centripetal obesity as well as folliculitis [103], while the effect on bone mineral density remains controversial [103,104]. Effects on hepatomegaly and hematological as well as immunological parameters are not evident [101,104].

Lysine supplementation restores lysine deficiency [104,108-110], but, unfortunately, long-term lysine supplementation failed to improve growth and to reduce infection frequency [104,110]. At high dosage (0.8-1.2 g/kg/d), lysine supplementation even induced gastrointestinal side-effects as abdominal cramps and diarrhoea [104,108]. Supplementation with homocitrulline to restore the lysine was unsuccessful [111].

Although arginine and ornithine supplementation were shown to prevent hyperammonemia [101,102,105,112], and to stimulate urea formation [101,105], long-term treatment effects remained inconclusive [101,105-107]. Various studies comparing different (combinations of) SAA supplementation regimens have not provided the optimal regimen for LPI [100,102,104,107], although all advise positively on citrulline. To conclude, long-term citrulline supplementation resulted in clinical improvement of LPI patients (level 4). The effect of other SAA supplementation regimens, especially on the long-term, is still unclear.

### Nonketotic hyperglycinemia

Nonketotic hyperglycinemia (NKH; McKusick 238300, 238310, and 238330) is characterized by a defective glycine cleavage system, resulting in excessive glycine concentrations in both blood and brain. The role of glycine as an excitatory agonist of the NMDA receptor in the cortex, and as an inhibitory neurotransmitter in the brain stem and spinal cord, may well explain NKH symptomatology, which is characterized primarily by intractable seizures and progressive brain damage. A glycine-restricted diet has been tried as well as benzoate and NMDA receptor antagonists, but without any clinical improvement, so no effective treatment exists.

To improve outcome, two different SAA supplementation regimens have been proposed without consistent positive results, including methionine to prevent toxic accumulation of glycine by supplying single carbon groups [113-115] and tryptophan to counteract the effect of elevated brain glycine on the NMDA receptor [116,117]. In a single case of NKH, tryptophan supplementation was reported to reduce hyperkinesia, explosive movements, and EEG abnormalities, as well as to improve verbal functioning and to increase the developmental quotient [116]. However, in another case of NKH, no behavioural improvements could be demonstrated on tryptophan supplementation [117]. To conclude, methionine supplementation in NKH patients is probably not effective (level 5), while the reported effects of tryptophan supplementation are inconsistent (level 5).

## Discussion

For most aminoacidopathies, dietary treatment includes natural protein restriction combined with a synthetic amino acid mixture devoid of the amino acids prior to the metabolic block or essential amino acid supplementation. Especially if the amino acid - that cannot be converted due to the enzymatic defect - is essential (e.g. phenylalanine in PKU), dietary management has proven to be effective [49,118,119]. On the contrary, if the nonconvertible amino acid is nonessential (e.g. tyrosine in Tyrosinemia type I), dietary treatment is less effective [120,121]. The present review addresses the applications, objectives, and treatment effects of additional SAA supplementation for purposes other than to overcome a deficiency of the amino acid that has become essential by the enzymatic defect. Before discussing the main conclusions in further detail, we will first address some methodological issues.

In the present review, supplementation of one or two amino acids that have become essential by the enzymatic defect in aminoacidopathies (e.g. tyrosine in PKU, arginine in urea cycle defects) is not being addressed. Such SAA supplementation has been widely reported on already. Supplementation of more than two amino acids is not being discussed. Various combinations of large neutral amino acids - except for phenylalanine - have since long been suggested to serve different biochemical treatment objectives in PKU, as has already been reviewed by Van Spronsen et al. [122]. Research on this subject is still being performed [123], but a study investigating all hypothesized biochemical treatment objectives of large neutral amino acid supplementation in PKU is still hoped-for. More recently, BCAA supplementation for urea cycle defects treated with alternate pathway treatment (sodium phenylbutyrate or phenylacetate/benzoate) has been proposed [124-126] to prevent strongly decreased blood BCAA concentrations [127,128] that have been shown to often precede metabolic decompensation [129]. Such supplementation is now being used in some clinics, but a beneficial effect has not been demonstrated yet [130]. The search strategy performed has been limited to articles written in English and German. Combined with the fact that especially rather dated articles could not all be obtained, this has posed the possibility of having missed some relevant articles.

The consideration of SAA supplementation in aminoacidopathies treated with natural protein restriction and synthetic amino acid mixtures is not always straightforward. First, treatment is not always based on comparable strategies. Even for one single aminoacidopathy, different SAA supplementation regimens can be considered, each with a different strategy. Also, a specific SAA supplementation regimen can be hypothesized to serve different strategies in one single aminoacidopathy. This complicates the formulation of uniform outcome measures.

Second, the need for SAA supplementation can either be posed directly by the disorder itself or secondary by its (dietary) treatment. If required due to dietary treatment, SAA supplementation mostly aims to restore a deficiency of specific amino acids. This seems to hold true especially for isoleucine. Of all amino acids, isoleucine has been found to be most critical for keratinocyte proliferation [23]. The importance of monitoring a deficiency of specific amino acids is exemplified in MMA, where low to very low concentrations of isoleucine and valine have been found to be widely present in patients, irrespective of the natural protein intake [25]. To restore specific amino acid deficiencies, three different challenges are experienced: 1) determining the minimal blood concentrations for individual amino acids; 2) monitoring amino acid levels; and 3) establishing optimal amino acid supplementation regimens.

The first challenge is especially applicable to HT1, as no data are available on the safe lower limits of blood phenylalanine concentrations. Theoretically, this can be an age-dependent parameter, related to velocity of (brain) development and growth.

Regarding the second challenge, careful monitoring of blood amino acid concentrations is crucial to timely detect deficiencies that are not yet clinically detectable, taking in mind that blood concentrations may not always reflect processes at a cellular level. Probably the most reliable method for detecting an amino acid deficiency is to determine whether the concentration of the specific amino acid either decreases (indicating that a deficiency is very likely), remains stable (indicating that a deficiency should be considered), or increases (indicating that a deficiency is unlikely) after a meal compared to the overnight fasting concentration [131]. Interpreting amino acid status can be particularly challenging if continuous tube feeding is given. Interpretation is further complicated by adherence issues. SAA are hard to administer (particularly in very small doses) and no studies address if patients actually consume them or take them in a way that utilization will be effective. Of course, an alternative strategy to detect amino acid deficiencies is the indicator amino acid oxidation method [8], but this method is used for research purposes rather than for clinical practice.

Regarding the third challenge, optimal treatment in response to deficiencies of offending precursor amino acids still remains to be investigated for most aminoacidopathies. Liberalization of dietary restrictions could prevent deficiencies of other essential nutrients besides amino acids, whereas, at the same time, it poses the risk of metabolic decompensation due to increased concentrations of other offending precursor amino acids. On the other hand, supplementation of SAA raises the question whether supplementation should be restricted to moments of measured deficiency or should be included in the daily amino acid mixtures.

Third, for many aminoacidopathies, evidence on the effects of SAA treatment is limited. The rarity of these disorders probably contributes in part to the fact that most evidence is restricted to case reports. Also, animal models, which allow for more invasive measurement and mechanism-based research, are not available for all disorders. However, even the data obtained from animal models are only to be used to some degree and, therefore, could not fully help us in this review to increase the level of evidence.

In conclusion, before application in routine clinical practice can be considered, for most SAA supplementation treatments in aminoacidopathies, further research is warranted, both in animal models and patients. It is the opinion of the authors that clinical research would first require measuring the need of the SAA to be supplemented in individual patients. If results show that for most patients, having the same disorder, the extra need of such amino acid is comparable, this could result in inclusion of the extra amount in the precursor-free amino acid mixtures or essential amino acid supplements. The arginine-enriched amino acid formula for GA-I or the isoleucine and valine enriched formula for acute MSUD management, as studied by Strauss et al., may be good examples [42,49]. To this purpose, well-designed clinical research is necessary with initially small and later on larger populations. Multi-center studies, possibly involving multiple countries, would be crucial in this regard.

The first treatment of inherited metabolic diseases was based on a change in nutritional intake, and this happened to be the first treatment ever of mental retardation. After that, many nutritional and medical interventions have been developed. Now, we might -again- enter a new era in which rather simple changes in nutritional intake may improve the outcome of patients with such devastating diseases. Such change may be elicited by supplementation of SAA, including both physiological and non-physiological amino acids, especially aiming at competition with toxic agents for entry into target organs (the brain in particular).

## Abbreviations

ADMA: Asymmetric dimethylarginine; BCAA: Branched-chain amino acids; CBS: Cystathionine β-synthase; GA: Gyrate atrophy; GA-I: Glutaric aciduria type I; GAA: Guanidinoacetate; GAMT: Guanidinoacetate methyltransferase; HCU: Homocystinuria; HHH: Hyperammonaemia-hyperornithinaemia-homocitrullinuria; HT1: Hereditary tyrosinemia type I; IVA: Isovaleric acid; IVG: N-isovalerylglycine; LPI: Lysinuric protein intolerance; MMA: Methylmalonic acidemia; MSUD: Maple syrup urine disease; NO: Nitrogen oxide; NKH: Nonketotic hyperglycinemia; NTBC: 2-(2-nitro-4-trifluoromethylbenzyl)-1,3-cyclohexanedione; OAT: Ornithine aminotransferase; PA: Propionic acidemia; PKU: Phenylketonuria; SAA: Single amino acid.

## Competing interests

MvR has received grants, consultancy fees, and advisory board fees from Merck Serono and Nutricia Research, speaker’s honoraria from Merck Serono, Nutricia Research, and Orphan Europe, and expert testimony fees from Merck Serono. FJvS has received research grants, advisory board fees, and speaker’s honoraria from Merck Serono and Nutricia Research. AM has received research grants, advisory board fees, and speaker’s honoraria from Merck Serono, Vitaflo International Ltd, and Nutricia Research. All other authors have declared not to have conflicts of interest.

## Authors’ contributions

FJvS, TGJD, and DvV were involved in the design of the systematic literature search. DvV performed the literature search and study selection, primarily supervised by FJvS and TGJD. DvV wrote the manuscript. FJvS and TGJD provided important contributions throughout the manuscript preparation process. MvR, MJdG, AM, and MRHF critically assessed the selected literature and made important contributions to the revision of the manuscript. All authors read and approved the final manuscript.

## Supplementary Material

Additional file 1 Suggested applications of SAA supplements in different aminoacidopathies treated with severe natural protein restriction and an amino acid mixture devoid of the offending precursor amino acids. Click here for file
