# Supplementary material for: Single amino acid supplementation in aminoacidopathies: a systematic review
Source: Orphanet J Rare Dis. 2014 Jan 13;9:7. doi: 10.1186/1750-1172-9-7 (PMC3895659; doi:10.1186/1750-1172-9-7)
Supplement: Additional file 1 — Suggested applications of SAA supplements in different aminoacidopathies treated with severe natural protein restriction and an amino acid mixture devoid of the offending precursor amino acids. [file 1750-1172-9-7-S1.pdf]

Supplement 1. Suggested applications of SAA supplements in different aminoacidopathies treated with severe natural protein restriction and an amino acid mixture devoid of the offending precursor amino acids.

Disorder	Treatment objective			Intervention	Biochemical effects	Clinical effects	LoE
	A	B	C				
IVA		Gly		Leu-load (25-125 mg/kg) combined with Gly (150-600 mg/kg) supplementation in IVA patients	Less increased plasma IVA concentrations than after unsupplemented Leu-load [10, 11, 13].<br>More increased urinary excretion of IVG [10-12] and associated metabolites [11] than after an unsupplemented Leu-load.<br>More increased urinary IVG excretion than after Leu-load with low-dosed Gly supplementation [16].<br>Less increased urinary excretion of β-OH hippuric acid and acetoacetic acid than after unsupplemented Leu-load [11].	No vomiting, as was observed after the unsupplemented Leu-load [10].<br>No nausea, as was observed after Leu-load with low-dosed Gly supplementation [16].	4-5
				Gly supplementation during ketoacidotic attack in IVA patients	Sharply decreased plasma IVA concentrations [15].<br>Increased urinary IVG excretion [13-15].<br>Thrombocytopenia and neutropenia resolved after three weeks [15].	Normalization of neurological condition after two weeks [15].<br>Decreased duration of clinical symptoms during ketoacidotic attack [10].	4-5
				Gly supplementation (50-600 mg/kg/d) in IVA patients under stable conditions	Increased plasma Gly concentrations [15, 17, 18] at increasing dosages of Gly supplementation [16].<br>Increased urinary IVG excretion [11, 15, 17, 18, 20].<br>Different response curves of urinary IVG excretion to increasing dosages of Gly supplementation [16].<br>Decreased urinary IVC excretion by 38% [20].	No untoward effects observed [11].<br>Increased lethargy and ataxia [17, 18].<br>Increased linear growth by 50% and weight gain by 156% [10].<br>No discernible neurologic disability [15].<br>Normal psychomotor development [15].<br>No ketoacidotic attacks despite confirmed infections [11, 15].<br>Decreased frequency of ketoacidotic attacks from 1.32/y to 0.31/y [19].	4-5
MMA	Ile+Val				No data on SAA supplementation.		
PA	Ile+Val				No data on SAA supplementation.		
		Gly		Gly supplementation (200 mg/kg, twice) and protein restriction during ketoacidotic attack in a PA patient	Increased blood ammonia concentrations [34].<br>Increased urinary tiglylglycine and propionylglycine excretion [34].<br>Unchanged urinary 3-hydroxypropionate, 2-methylcitrate, hippurate, and 4-hydroxyhippurate excretion [34].		5
GA-I			H-Arg	5% H-Arg added to the diet of GA-I mice	Reduced accumulation of cerebral Lys and glutaric acid to 50% and 41% of normal [40].	Increased 12-day survival rate from 20% to 80% [40].	animal
			Arg	Arg added to drinking water (2% of total dietary food intake) of GA-I mice	Reduced cerebral and hepatic accumulation of glutaric acid (74% and 68% of normal) [41].<br>Reduced Lys concentrations in cerebral and hepatic mitochondria to 85% and 52% of normal [41].		animal
				Arg fortified AA supplements (9% of total protein) in GA-I patients	Reduced plasma Lys concentrations and urinary 3-hydroxyglutarate excretion to 53% and 50% of values for patients treated with natural protein restriction [42].<br>Reduced calculated brain Lys influx and increased brain Arg influx to 56% and 154% of those for patients treated with natural protein restriction [42].	No alterations in age-specific weight and length curves [42].	3b
				Two types of Arg fortified AA supplements with different amounts of Arg, offered to GA-I patients	No significant relationship between Arg intake and plasma Lys-to-Arg ratios [43].	No significant relationship between Arg intake and neurological outcome [43]	3b
			Orn	Orn (5%) added to the diet of GA-I mice		Decreased 12-day survival rate in GA-I mice from 20% after 6-12 days to 0% after 5 days [39].	animal

MSUD			Ile+Val		No data on SAA supplementation.		
			NorLeu	NorLeu (5%) added to a high protein diet in MSUD mice	Decreased brain Leu concentrations to 80% [52].<br>Increased brain Glu, Asp, GABA, Trp and pyruvate concentrations [52].<br>Decreased brain Lys, Ala, α-ketoisocaproate and α-ketoglutarate concentrations [52].	Increased survival [52].<br>Higher neurological score [52].<br>Prolonged cage hanging [52].	animal
	Ile+Val			Ile (35-85 mg/kg/d) and Val (40-85 mg/kg/d) supplementation in a MSUD patient	Normalized blood Ile concentrations within 1 week [47].<br>Blood Hb reached normal levels [47].	Dermatitis resolved [47].<br>Diarrhea resolved [47].	5
				Ile (35 mg/kg/d) and Val (40 mg/kg/d) in a MSUD patient		Erythematous rash resolved [46].	5
				Ile (2.0-15 mg/kg) and Val (2.0-10 mg/kg) supplementation during acute metabolic decompensation in MSUD patients	Variable response on blood Ile and Val concentrations [51].<br>Further decreased blood Leu concentrations when blood Ile and Val concentrations reached normal values [51].		4
PKU	Trp			Trp supplementation (100 mg/kg/d) in PKU patients	Increased Trp concentrations in blood and CSF [66].<br>Unchanged Phe and Tyr concentrations in blood and CSF [66].<br>Increased 5-HIAA concentrations in CSF [66].<br>Increased HVA concentrations in CSF in 1 of 2 patients [66].	Increased vigilance if abnormal when untreated [66].	4
			MAIB	3% MAIB added to the diet of PKU mice	Reduced brain Phe concentrations by 14% [67].<br>Slightly reduced brain Tyr concentrations [67].<br>Reduced brain DOPAC, HVA, 3-MT, and 5-HIAA concentrations [67].		animal
			AIB	5% AIB added to the diet of PKU mice	Unchanged brain Phe concentrations [67].<br>Reduced brain Tyr, total BCAA and Met concentrations [67].<br>Reduced plasma Trp and Met concentratons [67].		animal
			NB	0.5% NB added to the diet of PKU mice	Reduced brain Phe concentrations by 27% [67].<br>Reduced brain Tyr, total BCAA and Met concentrations [67].<br>Reduced brain DA, 5-HT and 3-MT concentrations [67].		animal
			NL	5% NL added to the diet of PKU mice	Reduced brain Phe concentrations by 56% [67].<br>Reduced brain Tyr, total BCAA and Met concentrations [67].		animal
		Thr		Thr supplementation (appr. 50 mg/kg/d) in PKU patients	Reduced blood and urinary Phe concentrations [62].<br>Unchanged weight or height gain [62].		3b
		Gln		Gln supplementation (7.6 mM/kg) under fasting conditions in a PKU patient	Decreased urinary phenylpyruvic acid and phenyllactic acids excretion [59].<br>Unchanged blood Phe concentrations [59].		5
		Glu		Glu supplementation (7.6 mM/kg) under fasting conditions in a PKU patient	Decreased urinary phenylpyruvic acid and phenyllactic acids excretion [59].<br>Unchanged blood Phe concentrations [59].		5
		Asn		Asn supplementation (7.6 mM/kg) under fasting conditions in a PKU patient	Decreased urinary phenylpyruvic acid and phenyllactic acids excretion [59].<br>Unchanged blood Phe concentrations [59].		5
	Gln			Gln supplementation (200-1000 mg/kg/d) in PKU patients	No remarkable rise in blood glutamine concentrations [58].<br>No excessive urinary Gln or glutamic acid excretion [58].<br>Unchanged urinary PAG excretion [59].<br>Unchanged blood Gln concentrations [59].		4

HT1	Phe			Phe supplementation (20-40 mg/kg/d) in HT1 patients	Reduced incidence of blood Phe concentrations <35 µmol/L in the afternoon [70].		4
OAT deficiency		Lys		Lys supplementation (10 g/d) in OAT deficiency patients	Decreased blood Orn concentra-tions by 34% after one week [77].<br>Increased urinary Orn excretion by 775% [77].		4
				Lys supplementation (10-15 g/d) in OAT deficien-cy patients	Decreased blood Orn concentra-tions by 21-31% after 1-2 days [78].<br>Increased blood Lys concentrations by 381-414% [78].<br>Further decreased blood Orn con-centrations after increased Lys supplementation from 10 to 15 g/d [78].<br>Markedly increased urinary Lys and Orn excretion and moderately increased urinary Arg excretion [78].<br>Unchanged blood Arg and ammonia concentrations [78].	No side-effects observed [78].	4
	Pro			Pro supplementation (200-1000 mg/d) either alone or combined with pyri-doxine administra-tion in OAT deficiency patients	Unchanged blood Orn concentra-tions [79].<br>Increased blood Pro concentra-tions in three out of four patients [79].	Improved visual acuity and reduced refractive error in one patient [79].<br>Minimal chorioretinal dete-rioration in three out of four patients [79].	4
GAMT deficiency		Orn	Orn	Orn HCl supplementa-tion (100 mg/kg/d) and Arg restriction in a GAMT deficiency patient	Decreased blood Orn, Arg and GAA concentrations [86].<br>Decreased urinary GAA excretion [86].<br>Decreased Arg and GAA concentra-tions in CSF [86].	Decreased epileptogenic activity [86].<br>Improved contact with surrounding, alertness and motor activity [86].	5
				Orn HCl supplementa-tion (600-640 mg/kg/d) in a GAMT deficiency patient	Increased blood Orn concentrations [83].<br>Unchanged blood and urinary guanidinoacetate concentrations [83].		5
				Orn supplementation (100-800 mg/kg/d) in a GAMT deficiency patient	Unchanged cerebral creatine and GAA concentrations [89].<br>Decreased blood GAA concentra-tions [89].	Improved non-verbal IQ [89].<br>Increased language expres-sion from incomplete mono-syllabic utterances to senten-ces up to five words [89].	5
HCU		Cys			No data on SAA supplementation.		
			Arg	Arg (13.5 g) and Glu (11.5 g) supplementa-tion in an HCU patient	Unchanged blood homocystine concentrations [93].<br>Increased blood cystine, lysine, arginine, and ornithine concentra-tions [93].<br>Increased urinary homocystine and homocystine-cystine disulphide excretion [93].<br>Increased urinary Cys, Lys, Arg, and Orn concentrations [93].		5
	Arg*	Arg*	Arg*	Arg supplementation (2400 mg/d) in HCU patients	Increased blood Arg/ADMA ratio [92].<br>Unchanged blood homocysteine concentrations [92].<br>Decreased urinary 8-iso-$PGF_{2\alpha}$ excretion [92].	Enhanced endothelium – dependent vasodilation [92].	1b

Therapeutic objective A) correction of AA deficiency; B) prevention of toxic accumulation of specific substrates prior to the metabolic block; C) competition with toxic agents for entry into target organs.

LoE: level of evidence (as determined by the criteria from www.cebm.net); AIB: 2-aminoisobutyrate; Ala: alanine; Arg: arginine; Asn: asparagines; Citr: citrulline; Cys: cyst(e)ine; DA: dopamine; DOPAC: 3,4-dihydroxyphenylacetic acid; GAA: guanidinoacetate; GABA: gamma-aminobutyric acid; GH: growth hormone; Gln: glutamine; Glu: glutamate; Gly: glycine; H-Arg: homoarginine; H-Citr: homocitrulline; 5-HIAA: 5-hydroxyindoleacetic acid; 5-HT: serotonin; HVA: homovanillic acid; Ile: isoleucine; Leu: leucine; Lys: lysine; MAIB: N-methyl-aminoisobutyrate; Met: methionine; 3-MT: 3-methoxytyramine; NB: 2-aminonorbornane; NorLeu: norleucine; Orn: ornithine; PAG: phenylacetylglutamine; Phe: phenylalanine; Pro: proline; Thr: threonine; Trp: tryptophan; Val: valine; VMA: vanillylmandelic acid

* treatment objective is unclear
